# Some New Results on the Gaussian Wiretap Feedback Channel

**DOI:** 10.3390/e21090817

**Published:** 2019-08-21

**Authors:** Chenxu Wei, Linman Yu, Bin Dai

**Affiliations:** 1 School of Information Science and Technology, Southwest Jiaotong University, Chengdu 611756, China; 2 School of Economics and Management, Chengdu Textile College, Chengdu 611731, China

**Keywords:** Gaussian wiretap channel, noiseless feedback, Schalkwijk–Kailath scheme, secrecy capacity

## Abstract

In this paper, the Gaussian wiretap feedback channel is revisited, and some new results on its secrecy capacity are obtained. To be specific, first, we show that the Schalkwijk–Kailath (SK) feedback scheme, which achieves the secrecy capacity of the degraded Gaussian wiretap feedback channel, also achieves the secrecy capacity of the non-degraded Gaussian wiretap feedback channel. Second, applying the existing secret key-based feedback schemes to Gaussian wiretap feedback channels, we derive some new lower bounds on the secrecy capacities of these models. Finally, we compare the performances of the above feedback schemes in the degraded and non-degraded Gaussian wiretap feedback channels and show which feedback scheme performs better for these channel models.

## 1. Introduction

In recent years, mobile wireless communication has been widely used and has become an essential part in people’s daily life. Due to the broadcast nature of wireless communications, the private information in people’s wireless mobile devices (such as bank card information, energy pricing messages, e-health data, and password messages) is more vulnerable to eavesdropping. Physical layer security (PLS), realizing secure communication over wireless channels by information-theoretic approaches, is shown to be an effective way to prevent information eavesdropping. The research on PLS in communication systems started from Wyner’s outstanding work on the degraded wiretap channel (DWTC) [1], where a transmitter broadcasts its message *M* over *N* channel uses to a legitimate receiver and an eavesdropper via a degraded broadcast channel, and the perfect secrecy is guaranteed if the information leakage rate $\frac{1}{N}I\left(M;Z^{N}\right)$, where $Z^{N}$ denotes the received output at the eavesdropper, vanishes as the transmitted codeword length *N* tends to infinity (Here note that the perfect secrecy defined in [1] is in fact weak secrecy. Another definition of the perfect secrecy is strong secrecy, which is defined as the information leakage $I(M;Z^{N})$ at the wiretapper vanishes as *N* tends to infinity.).The secrecy capacity, defined as the channel capacity under the weak secrecy constraint, was established in [1]. Subsequently, the work in [2] generalized the DWTC [1] by considering a general broadcast channel and the transmission of a common message, which is allowed to be decoded by both the legitimate receiver and the eavesdropper. The capacity results of [1] and [2] indicated that for the wiretap channel (WTC) and its extended model, the positive secrecy rates are guaranteed only if the legitimate receiver’s channel is less noisy than the wiretapper’s channel. Thus, it is natural to ask the following two questions:

- (1) How can a positive secrecy rate be achieved if the eavesdropper’s channel is less noisy than the legitimate receiver’s channel?
- (2) If the eavesdropper’s channel is noisier than the legitimate receiver’s channel, can the secrecy rate be further enhanced beyond the secrecy capacity?

The schemes that exploit artificial noise-aided cooperative jamming [3,4,5] and channel feedback address the above two questions. However, in some circumstances, such as Internet of Things (IoT) systems, artificial noise-aided cooperative jamming may not be suitable since the IoT devices have significant energy constraints [6,7], and hence, channel feedback is of particular interest in such circumstances.

The role of channel feedback in PLS of communication systems was first studied in [8], where the pioneering work [1] was re-visited by considering the case that the legitimate receiver can send its received channel outputs back to the transmitter via a noiseless feedback channel, which is not known by the eavesdropper. Since the transmitter also knows the legitimate receiver’s channel output via the noiseless feedback channel, the work in [8] showed that generating the secret key from the legitimate receiver’s channel output and using it to encrypt the transmitted message help to increase the secrecy capacity of the WTC. Furthermore, the work in [8] showed that such a secret key-based feedback scheme achieves the secrecy capacity of the DWTC with noiseless feedback, which implies that it is an optimal feedback scheme for the DWTC. Here, note that in [8], the feedback channel only transmits the legitimate receiver’s channel output, and what happens if the channel can transmit anything as the legitimate receiver wishes? The work in [9] investigated this case and pointed out that directly transmitting pure random bits instead of the legitimate receiver’s channel output over the noiseless feedback channel may perform even better. [9] further showed that transmitting pure random bits performs better than transmitting the legitimate receiver’s channel output if the rate of the pure random bits is larger than that of the secret key generated from the legitimate receiver’s channel output, and vice versa. Later, the work in [10] extended the WTC with rate-limited feedback [9] to a broadcast case, where one secret message is sent to two legitimate receivers via a general broadcast wiretap channel, and two legitimate receivers independently send their secret keys to the transmitter via two noiseless feedback channels. Encrypting the transmitted message for its intended legitimate receiver by the corresponding secret key and using time-sharing between these two encrypted messages, the work in [10] derived an achievable secrecy rate for this extended model and showed that these secret keys help to increase the achievable secrecy rate (lower bound on the secrecy capacity) of the same model without feedback [11]. Other related works on the feedback channels with secrecy constraints include [12,13,14,15,16], where the channel state was introduced into various feedback channel models in the presence of an eavesdropper.

Here, note that the feedback schemes mentioned above mainly focus on generating secret keys from the feedback. Recently, exploiting other usages of feedback has attracted considerable attention. To be specific, the work in [17] showed that for feedback communication systems, a better use of the channel output feedback is to produce not only a secret key, but also a helping message from it, and such a helping message improves the legitimate receiver’s decoding performance. Later, the works in [18] and [19] further applied the scheme of [17] to the state-dependent WTC with and without the action encoder, respectively. Moreover, the work in [20] found that the classical Schalkwijk–Kailath (SK) scheme [21] achieving the capacity of the Gaussian channel with feedback also achieved the secrecy capacity of the Gaussian wiretap channel with feedback. Furthermore, the work in [22] investigated the finite-order autoregressive moving average (ARMA) Gaussian wiretap channel with noiseless feedback. A variation of the SK scheme was proposed to achieve the secrecy capacity, which equals the capacity of the same model without the secrecy constraint.

**Figure 1:**
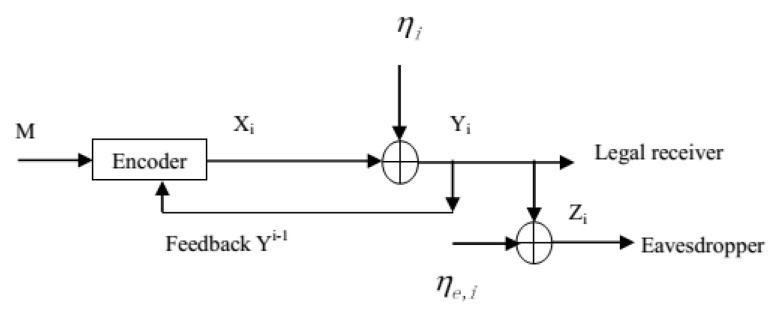
The degraded Gaussian wiretap feedback channel.

**Figure 2:**
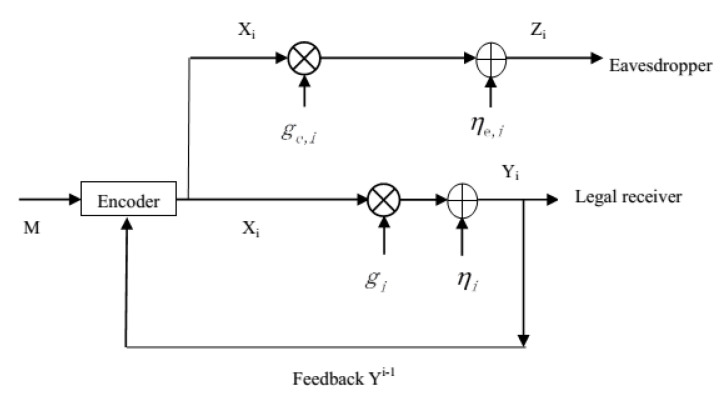
The non-degraded Gaussian wiretap feedback channel.

In this paper, we revisit the Gaussian wiretap feedback channel [20] (see Figure 1), and would like to answer the following questions:

- (1) In [20], the secrecy capacity of the degraded Gaussian wiretap feedback channel was derived, and it equaled the capacity of the same model without the secrecy constraint. Does this still hold for the non-degraded Gaussian wiretap feedback channel (see Figure 2), i.e., does the secrecy capacity of the non-degraded Gaussian wiretap feedback channel equal the capacity of the same model without the secrecy constraint?
- (2) For the already existing feedback schemes such as the secret key based feedback scheme [8], the improved secret key-based feedback scheme [17], and the SK feedback scheme [20,21], which one performs the best for the Gaussian wiretap feedback channel?

The main contribution of this paper is as follows:

- (1) We derive the secrecy capacity of the non-degraded Gaussian wiretap feedback channel and show that it also equals the capacity of the same model without the secrecy constraint.
- (2) In [8], it was shown that the secret key-based feedback scheme was optimal for the discrete memoryless DWTC. However, this is not true for the degraded Gaussian wiretap feedback channel, i.e., in this paper, we show that the secret key-based feedback scheme only achieves a lower bound on the secrecy capacity of the degraded Gaussian wiretap feedback channel. Hence, for the degraded Gaussian wiretap feedback channel, the SK feedback scheme performs the best. In addition, in this paper, we show that for the non-degraded Gaussian wiretap feedback channel, the improved secret key-based feedback scheme performs as well as the SK scheme, and both of them perform better than the secret key-based feedback scheme.

This paper is organized as follows. Section 2 shows the capacity result on the non-degraded Gaussian wiretap feedback channel and its proof. Section 3 shows the performances of the secret key-based feedback scheme, the improved secret key-based feedback scheme, and the SK feedback scheme in the Gaussian wiretap feedback channel. Final conclusions are presented in Section 4.

## 2. The Non-Degraded Gaussian Wiretap Feedback Channel

In the remainder of this paper, random variables (RVs), their realizations, and alphabets are denoted by uppercase letters, lowercase letters, and calligraphic letters, respectively. Random vectors and their realizations are written in a similar way. For example, *Y* denotes an RV, and *y* denotes the value of a realization in the alphabet Y. Similarly, $Y^{N}$ denotes a random vector $\left(Y_{1},\ldots ,Y_{N}\right)$, and $y^{N}=\left(y_{1},\ldots ,y_{N}\right)$ denotes the value of a realization in $Y^{N}$ (the $N^{\mathrm{th}}$ Cartesian power of Y). Moreover, for simplicity, the probability Pr{X=x} is denoted by P(x), and in the remainder of this paper, the base of the log function is taken to be two.

For the non-degraded Gaussian wiretap feedback channel (see Figure 2), the transmitted message *M* is uniformly drawn from the set M={1,2,…,|M|}. The channel input and outputs at time i∈{1,2,…,N} satisfy:

(1) $$\begin{array}{cc} & Y_{i}=g_{i}X_{i}+\eta_{i},\;\;\;\;\;Z_{i}=g_{e,i}X_{i}+\eta_{e,i}, \end{array}$$

where $X_{i}$ is the channel input subject to an average power constraint *P*, $Y_{i}$ and $Z_{i}$ are channel outputs respectively at the legitimate receiver and the eavesdropper, $g_{i}=g$ and $g_{e,i}=g_{e}$ are the gains of the legitimate receiver’s channel and the eavesdropper’s channel, respectively, and $\eta_{i}$ and $\eta_{e,i}$ are independent Gaussian noises and are i.i.d. across the time index *i*. Moreover, $\eta_{i}∼N\left(0,\sigma^{2}\right)$, $\eta_{e,i}∼N\left(0,\sigma_{e}^{2}\right)$ and the noises $\eta_{i}$, $\eta_{e,i}$ are independent of the transmitted message *M*, and the $i^{\mathrm{th}}$ channel input $X_{i}$ is a stochastic function of the message *M* and the channel output feedback $Y^{i-1}$.

The legitimate receiver produces $\hat{M}=\psi \left(Y^{N}\right)$, where ψ is the legitimate receiver’s decoding function, and the decoding error probability is denoted by:

(2) $$P_{e}=\frac{1}{\left|M\right|}\sum_{m\in M}Pr\left\{\psi \left(y^{N}\right)\neq m|m\;\;\mathrm{sent}\right\}.$$

Let:

(3) $$\Delta =\frac{1}{N}H\left(M|Z^{N}\right)$$

be the eavesdropper’s equivocation rate of the message *M*. Given a non-negative number *R*, if for any ϵ>0, there exists a pair of encoder and decoder such that:

(4) $$\begin{array}{cc} & \frac{\log|M|}{N}\geq R-\epsilon ,\;\;\Delta \geq R-\epsilon ,\;\;P_{e}\leq \epsilon , \end{array}$$

*R* is achievable under the weak secrecy constraint. The secrecy capacity $C_{s-f}$ is the supremum over all achievable weak secrecy rates, and it will be given in the following Theorem 1.

**Theorem** **1.** *The secrecy capacity $C_{s-f}$ of the non-degraded Gaussian wiretap feedback channel is given by:*

(5) $$\begin{array}{cc} & C_{s-f}=\frac{1}{2}\log\left(1+\frac{g^{2}P}{\sigma^{2}}\right). \end{array}$$

**Remark** **1.** *Here, note that in the model of Figure 2, the eavesdropper’s channel may be less noisy than the legitimate receiver’s. Theorem 1 indicates that even if the eavesdropper’s channel is less noisy than the legitimate receiver’s, the perfect secrecy can still be achieved without loss of the transmission rate, i.e., the secrecy capacity equals the legitimate receiver’s channel capacity.*

**Proof.**  First, remember that the capacity of the legitimate receiver’s channel is $\frac{1}{2}\log\left(1+\frac{g^{2}P}{\sigma^{2}}\right)$, and it is obtained by substituting (1) into maxI(X;Y) and using the fact that the maximum is achieved if *X* is Gaussian distributed with zero mean and variance *P*. Then, the converse of Theorem 1 follows from the fact that feedback does not increase the capacity of the legitimate receiver’s channel and $C_{s-f}$ cannot exceed the capacity of the legitimate receiver’s channel with feedback, i.e., $C_{s-f}\leq \frac{1}{2}\log\left(1+\frac{g^{2}P}{\sigma^{2}}\right)$. Now, it remains to show the achievability of $C_{s-f}$; see the following.

From (1), we know that the input and output of the legitimate receiver’s channel satisfy:

(6) $$\begin{array}{cc} & Y_{i}=gX_{i}+\eta_{i}. \end{array}$$

Notice that (6) can be re-written as:

(7) $$\begin{array}{cc} & Y_{i}^{\prime }=X_{i}+\eta_{i}^{\prime }, \end{array}$$

where $Y_{i}^{\prime }=\frac{Y_{i}}{g}$ and $\eta_{i}^{\prime }=\frac{\eta_{i}}{g}$. Now, the legitimate receiver’s channel is equivalent to a new Gaussian channel with input $X_{i}$, output $Y_{i}^{\prime }$, and channel noise $\eta_{i}^{\prime }∼N\left(0,\sigma^{{}^{\prime }2}=\frac{\sigma^{2}}{g^{2}}\right)$. Then, we describe the SK scheme for this equivalent channel as follows.

The message *M* takes values in the set $M=\{1,2,\ldots ,2^{NR}\}$. Divide the overall interval [−0.5,0.5] into $2^{NR}$ equally-spaced sub-intervals, and the center of each sub-interval is mapped to a message value in M. Let θ be the center of the sub-interval with respect to (w.r.t.) the choosing message *M*. At Time 1, the transmitter sends:

(8) $$\begin{array}{cc} & X_{1}=\theta \alpha , \end{array}$$

where $\alpha =\sqrt{\frac{P+\sigma^{{}^{\prime }2}}{\sigma^{{}^{\prime }2}}}=\sqrt{\frac{g^{2}P+\sigma^{2}}{\sigma^{2}}}$. Upon receiving the output $Y_{1}=hX_{1}+\eta_{1}$, the legitimate receiver obtains $Y_{1}^{\prime }=\frac{Y_{1}}{g}=X_{1}+\frac{\eta_{1}}{g}=X_{1}+\eta_{1}^{\prime }$ and computes:

(9) $$\begin{array}{cc} & {\hat{\theta }}_{1}=\frac{Y_{1}^{\prime }}{\alpha }=\theta +\frac{\eta_{1}^{\prime }}{\alpha } \end{array}$$

as an estimation of θ at Time 1. At time *i* (i∈{2,3,…,N}), the transmitter sends:

(10) $$\begin{array}{cc} & X_{i}=\alpha_{i}\left(\theta -{\hat{\theta }}_{i-1}\right)=-\alpha_{i}\frac{\sum_{j=1}^{i-1}\alpha_{j}\eta_{j}^{\prime }}{\sum_{j=1}^{i-1}\alpha_{j}^{2}}, \end{array}$$

where $\alpha_{i}=\sqrt{\frac{P}{\sigma^{{}^{\prime }2}}}\alpha^{i-1}=\sqrt{\frac{g^{2}P}{\sigma^{2}}}\alpha^{i-1}$ for i∈{2,3,…,N}. Upon receiving the output $Y_{i}=gX_{i}+\eta_{i}$, the legitimate receiver obtains $Y_{i}^{\prime }=\frac{Y_{i}}{g}=X_{i}+\frac{\eta_{i}}{g}=X_{i}+\eta_{i}^{\prime }$ and computes:

(11) $$\begin{array}{cc} & {\hat{\theta }}_{i}=\theta +\frac{\sum_{j=1}^{i}\alpha_{j}\eta_{j}^{\prime }}{\sum_{j=1}^{i}\alpha_{j}^{2}} \end{array}$$

as an estimation of θ at time *i*. In [21], it was shown that the decoding error probability $P_{e}$ (the probability of ${\hat{\theta }}_{N}$ not belonging to the sub-interval of the choosing message *M*) of this proposed scheme doubly-exponentially decays to zero for sufficiently large *N* and $R\leq \frac{1}{2}\log\left(1+\frac{P}{\sigma^{{}^{\prime }2}}\right)=\frac{1}{2}\log\left(1+\frac{g^{2}P}{\sigma^{2}}\right)$. Hence, letting $R=\frac{1}{2}\log\left(1+\frac{g^{2}P}{\sigma^{2}}\right)$, for a given ϵ, $\frac{\log|M|}{N}\geq \frac{1}{2}\log\left(1+\frac{g^{2}P}{\sigma^{2}}\right)-\epsilon$ and $P_{e}\leq \epsilon$ are satisfied by using the above proposed SK scheme. Then, it remains to show $\Delta \geq \frac{1}{2}\log\left(1+\frac{g^{2}P}{\sigma^{2}}\right)-\epsilon$, and the proof is given as follows.

(12) $$\begin{array}{l} \Delta =\frac{1}{N}H\left(M|Z^{N}\right)\overset{\left(1\right)}{=}\frac{1}{N}H\left(M|g_{e}X_{1}+\eta_{e,1},\ldots ,g_{e}X_{N}+\eta_{e,N}\right)\\ \overset{\left(2\right)}{=}\frac{1}{N}H\left(M|g_{e}\theta \alpha +\eta_{e,1},g_{e}\left(\frac{-\alpha_{2}\eta_{1}^{\prime }}{\alpha_{1}}\right)+\eta_{e,2},\ldots ,g_{e}\left(-\alpha_{N}\frac{\sum_{j=1}^{N-1}\alpha_{j}\eta_{j}^{\prime }}{\sum_{j=1}^{N-1}\alpha_{j}^{2}}\right)+\eta_{e,N}\right)\\ \geq \frac{1}{N}H\left(M|g_{e}\theta \alpha +\eta_{e,1},g_{e}\frac{-\alpha_{2}\eta_{1}^{\prime }}{\alpha_{1}}+\eta_{e,2},\ldots ,g_{e}\left(-\alpha_{N}\frac{\sum_{j=1}^{N-1}\alpha_{j}\eta_{j}^{\prime }}{\sum_{j=1}^{N-1}\alpha_{j}^{2}}\right)+\eta_{e,N},\eta_{1}^{\prime },\ldots ,\eta_{N}^{\prime },\eta_{e,2},\ldots ,\eta_{e,N}\right)\\ =\frac{1}{N}H\left(\theta |g_{e}\theta \alpha +\eta_{e,1},\eta_{1}^{\prime },\ldots ,\eta_{N}^{\prime },\eta_{e,2},\ldots ,\eta_{e,N}\right)\\ \overset{\left(3\right)}{=}\frac{1}{N}H\left(\theta |g_{e}\theta \alpha +\eta_{e,1}\right)\\ \overset{\left(4\right)}{=}\frac{1}{N}\left(H\left(\theta \right)+h\left(\eta_{e,1}\right)-h\left(g_{e}\theta \alpha +\eta_{e,1}\right)\right)\\ \overset{\left(5\right)}{=}\frac{1}{N}\left(NR+h\left(\eta_{e,1}\right)-h\left(g_{e}\theta \alpha +\eta_{e,1}\right)\right)\\ \overset{\left(6\right)}{=}\frac{1}{N}\left(NR+\frac{1}{2}\log\left(2\pi e\sigma_{e}^{2}\right)-h\left(g_{e}\theta \alpha +\eta_{e,1}\right)\right)\\ \overset{\left(7\right)}{\geq }\frac{1}{N}\left(NR+\frac{1}{2}\log\left(2\pi e\sigma_{e}^{2}\right)-\frac{1}{2}\log\left(2\pi e\left(\alpha^{2}g_{e}^{2}Var\left(\theta \right)+\sigma_{e}^{2}\right)\right)\right)\\ \overset{\left(8\right)}{=}\frac{1}{N}\left(NR+\frac{1}{2}\log\left(2\pi e\sigma_{e}^{2}\right)-\frac{1}{2}\log\left(2\pi e\left(\alpha^{2}g_{e}^{2}\frac{1}{12}+\sigma_{e}^{2}\right)\right)\right)\\ =R-\frac{1}{2N}\log\left(1+\frac{\alpha^{2}g_{e}^{2}}{12\sigma_{e}^{2}}\right)\\ \overset{\left(9\right)}{=}\frac{1}{2}\log\left(1+\frac{g^{2}P}{\sigma^{2}}\right)-\frac{1}{2N}\log\left(1+\frac{\left(g^{2}P+\sigma^{2}\right)g_{e}^{2}}{12\sigma_{e}^{2}\sigma^{2}}\right), \end{array}$$

where (1) follows from (1), (2) follows from (8) and (10), (3) follows from the fact that $\left(\eta_{1},\ldots ,\eta_{N},\eta_{e,2},\ldots ,\eta_{e,N}\right)$ are independent of θ, $g_{e}\theta \alpha +\eta_{e,1}$, and $\eta_{i}^{\prime }=\frac{\eta_{i}}{g}$, (4) follows from θ being independent of $\eta_{e,1}$, (5) follows from the fact that *M* is uniformly distributed over $M=\{1,2,\ldots ,2^{NR}\}$, (6) follows from $h\left(\eta_{e,1}\right)=\frac{1}{2}\log\left(2\pi e\sigma_{e}^{2}\right)$, (7) follows from the fact that $h\left(X\right)\leq \frac{1}{2}\log\left(2\pi eVar\left(X\right)\right)$, where the equality holds if *X* is Gaussian distributed, (8) follows from the fact that the variance of θ is $\frac{1}{12}$ while *N* tends to infinity (see a similar argument in [20]), and (9) is from the definitions $R=\frac{1}{2}\log\left(1+\frac{g^{2}P}{\sigma^{2}}\right)$ and $\alpha =\sqrt{\frac{g^{2}P+\sigma^{2}}{\sigma^{2}}}$. Finally, choosing sufficiently large *N*, $\Delta \geq \frac{1}{2}\log\left(1+\frac{g^{2}P}{\sigma^{2}}\right)-\epsilon$ is proven. The proof of Theorem 1 is complete. □

**Remark** **2.** *From the above proof of Theorem 1 (especially the inequality below Step (2) of (12)), we see that even if the eavesdropper obtains his/her own channel noises of all time indexes except Time 1 and knows the legitimate receiver’s channel noises of all time indexes, the weak secrecy can still be guaranteed with the transmission rate $R=\frac{1}{2}\log\left(1+\frac{g^{2}P}{\sigma^{2}}\right)$, and the intuition behind this fact is given as follows. The transmitter transmits the original message M only at the first transmission (see (8) and (10)), and then, the transmissions after the first one combine only channel noises in the previous transmissions. Since the information leakage occurs only in the first transmission, the information leakage rate $\frac{1}{N}I\left(M;Z^{N}\right)$ vanishes as the codeword length N tends to infinity.*

*The equivocation analysis (see (12)) of the proof of Theorem 1 also indicates that if the eavesdropper knows the legitimate receiver’s channel noises of all time indexes, i.e., $\eta_{1}^{\prime }$,…,$\eta_{N}^{\prime }$, he/she also obtains the channel feedback from Time 2–N (i.e., $Y_{2}$,…,$Y_{N}$) due to the reason that for i∈{2,3,…,N}, $X_{i}$ is only a combination of the channel noises in the previous transmissions (see (10)) and $Y_{i}=gX_{i}+g\eta_{i}^{\prime }$. Then, we can conclude that even if the channel output feedback $Y_{2}$,…,$Y_{N}$ is obtained by the eavesdropper, the weak secrecy can still be guaranteed. However, we should note that if the eavesdropper knows $Y_{1}$ and $\eta_{1}^{\prime }$, he/she also obtains the transmitted message since $Y_{1}=gX_{1}+g\eta_{1}^{\prime }$ and $X_{1}=\theta \alpha$, which implies that the weak secrecy cannot be guaranteed for this case.*

## 3. Comparison of the Already Existing Feedback Schemes for the Gaussian Wiretap Feedback Channel

In this section, we compare the performances of the secret key-based feedback scheme [8], the improved secret key-based feedback scheme [17], and the SK feedback scheme [21] in the Gaussian wiretap feedback channel.

### 3.1. Comparison of the Feedback Schemes for the Degraded Gaussian Wiretap Feedback Channel

For the degraded Gaussian wiretap feedback channel (see Figure 1), at time *i* (i∈{1,2,…,N}), the channel input and outputs are given by:

(13) $$\begin{array}{cc} & Y_{i}=X_{i}+\eta_{i},\;\;Z_{i}=X_{i}+\eta_{i}+\eta_{e,i}, \end{array}$$

where $X_{i}$ is the channel input with power constraint *P*, $Y_{i}$ and $Z_{i}$ are channel outputs respectively at the legitimate receiver and the eavesdropper, and $\eta_{i}∼N\left(0,\sigma^{2}\right)$ and $\eta_{e,i}∼N\left(0,\sigma_{e}^{2}\right)$ are independent channel noises and are i.i.d. across the time index *i*. The channel encoder, decoder, and the achievable secrecy rate are defined the same as in Section 2. The following Theorem 2 [20] determines the secrecy capacity $C_{s-f}^{d}$ of the degraded Gaussian wiretap feedback channel; see the following.

**Theorem** **2.** *The secrecy capacity $C_{s-f}^{d}$ of the degraded Gaussian wiretap feedback channel is given by:*

(14) $$\begin{array}{cc} & C_{s-f}^{d}=\frac{1}{2}\log\left(1+\frac{P}{\sigma^{2}}\right). \end{array}$$

**Remark** **3.** *Here, note that $C_{s-f}^{d}$ is achieved by using the SK feedback scheme. Theorem 2 indicates that for the degraded Gaussian wiretap feedback channel, the perfect secrecy can be achieved without loss of the transmission rate, i.e., the secrecy capacity equals the capacity of the legitimate receiver’s channel.*

**Proof.**  See [20]. □

In [8], it has been shown that the secrecy capacity $C_{s}^{*}$ of the discrete memoryless degraded wiretap feedback channel can be achieved by using the secret key-based feedback scheme (here, note that for the degraded wiretap feedback channel, the work in [17] showed that the improved secret key-based feedback scheme reduces to the original secret key-based feedback scheme [8]), and it is given by:

(15) $$\begin{array}{cc} & C_{s}^{*}=\max_{P(x)}\min\left\{I\left(X;Y\right),H\left(Y|Z\right)\right\}, \end{array}$$

where X→Y→Z. However, we should note that the capacity formula in (15) is only an achievable secrecy rate for the degraded Gaussian wiretap feedback channel, and this is because the converse of H(Y|Z) in (15) does not hold for the Gaussian case. To be specific, first, note that the term H(Y|Z) in (15) follows from:

(16) $$\begin{array}{ccc} R-\epsilon & \leq & \frac{1}{N}H\left(M|Z^{N}\right)\\ & = & \frac{1}{N}\left(H\left(M|Z^{N}\right)-H\left(M|Y^{N},Z^{N}\right)+H\left(M|Y^{N},Z^{N}\right)\right)\\ & \leq & \frac{1}{N}\left(I\left(M;Y^{N}|Z^{N}\right)+\delta \left(\epsilon \right)\right)\\ & \overset{\left(a\right)}{\leq } & \frac{1}{N}\left(H\left(Y^{N}|Z^{N}\right)+\delta \left(\epsilon \right)\right)\\ & \leq & \frac{1}{N}\left(\sum_{i=1}^{N}H\left(Y_{i}|Z_{i}\right)+\delta \left(\epsilon \right)\right)\\ & \overset{\left(b\right)}{=} & H\left(Y_{J}|Z_{J},J\right)+\frac{1}{N}\delta \left(\epsilon \right)\\ & \overset{\left(c\right)}{\leq } & H\left(Y|Z\right)+\frac{1}{N}\delta \left(\epsilon \right), \end{array}$$

and letting ϵ→0, where (a) follows from $I\left(M;Y^{N}|Z^{N}\right)\leq H\left(Y^{N}|Z^{N}\right)$, (b) follows from *J* being uniformly distributed over {1,2,…,N} and it being independent of $Y^{N}$ and $Z^{N}$, and (c) follows from the definitions $Y≜Y_{J}$ and $Z≜Z_{J}$. Next, from (16), we can check that for the Gaussian case, Step (a) of (16) does not hold due to the fact that the differential conditional entropy $h(Y^{N}|Z^{N},M)$ may be a negative number. Finally, substituting X∼N(0,P) and (13) into (15), a lower bound $R_{s-f}^{*}$ on the secrecy capacity $C_{s-f}^{d}$ is obtained, and it is given by the following Corollary 1.

**Corollary** **1.** *A lower bound $R_{s-f}^{*}$ on the secrecy capacity $C_{s-f}^{d}$ of the degraded Gaussian wiretap feedback channel is given by:*

(17) $$\begin{array}{cc} & R_{s-f}^{*}=\min\left\{\frac{1}{2}\log\left(1+\frac{P}{\sigma^{2}}\right),\frac{1}{2}\log\left(\frac{2\pi e\sigma_{e}^{2}\left(P+\sigma^{2}\right)}{P+\sigma^{2}+\sigma_{e}^{2}}\right)\right\}. \end{array}$$

**Figure 3:**
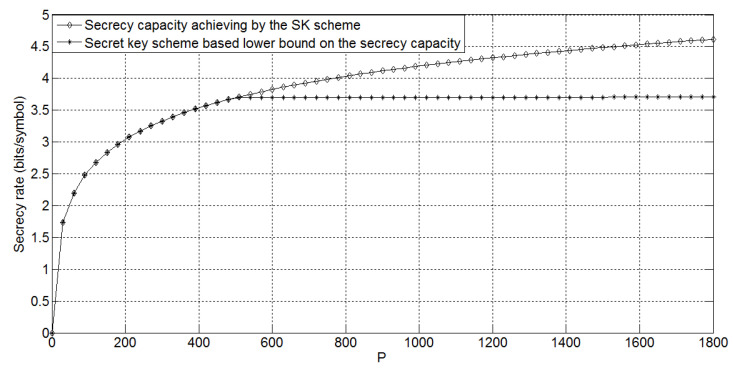
The capacity results on the degraded Gaussian wiretap feedback channel for $\sigma^{2}=3$, $\sigma_{e}^{2}=10$, and *P* taking values in [0,1800]. SK, Schalkwijk–Kailath.

Comparing $R_{s-f}^{*}$ in Corollary 1 with $C_{s-f}^{d}$ in Theorem 2, we can conclude that for the degraded Gaussian wiretap feedback channel, the secret key-based feedback scheme performs no better than the SK scheme. The following Figure 3 shows the gap between the lower bound $R_{s-f}^{*}$ and the secrecy capacity $C_{s-f}^{d}$ for $\sigma^{2}=3$, $\sigma_{e}^{2}=10$, and *P* taking values in [0,1800]. It is easy to see that the gap is increasing while the power *P* is increasing.

### 3.2. Comparison of the Feedback Schemes for the Non-Degraded Gaussian Wiretap Feedback Channel

In Section 2, we showed that the secrecy capacity of the non-degraded Gaussian wiretap feedback channel equals the legitimate receiver’s channel capacity. For comparison, in this subsection, we calculate the lower bounds constructed by the secret key-based feedback scheme [8] and the improved secret key-based feedback scheme [17]; see the following.

First, note that in [8], it has been shown that for the discrete memoryless non-degraded wiretap feedback channel, a lower bound $R_{s-f}^{**}$ on the secrecy capacity, which is constructed by the secret key-based feedback scheme, is given by:

(18) $$\begin{array}{cc} & R_{s-f}^{**}=\max_{P(x)}\min\left\{{\left[I\left(X;Y\right)-I\left(X;Z\right)\right]}^{+}+H\left(Y|X,Z\right),I\left(X;Y\right)\right\}, \end{array}$$

where Y→X→Z and ${\left[x\right]}^{+}=\max\left\{0,x\right\}$. The intuition behind (18) is given as follows. The feedback channel output is used to generate a secret key shared between the legitimate parties, and this key is completely unknown by the wiretapper. Moreover, the transmitted message *M* is divided into two parts $M_{1}$ and $M_{2}$, where $M_{1}$ is encoded the same as the message in [1], and $M_{2}$ is encrypted by the secret key generated from the feedback. Then, the total secrecy rate also consists of two parts: one equals I(X;Y)−I(X;Z), which is the same as the secrecy capacity of the wiretap channel [1], and the other equals H(Y|X,Z), which is the rate of the secret key. In addition, note that the total secrecy rate cannot exceed the channel capacity I(X;Y) of the legitimate parties, and hence, the lower bound in (18) is obtained. Then, substituting X∼N(0,P) and (1) into (18), a lower bound $R_{s-f}^{non*}$ on the secrecy capacity $C_{s-f}$ is obtained, and it is given by the following Corollary 2.

**Corollary** **2.** *A lower bound $R_{s-f}^{non*}$ on the secrecy capacity $C_{s-f}$ of the non-degraded Gaussian wiretap feedback channel is given by:*

(19) $$\begin{array}{cc} & R_{s-f}^{non*}=\min\left\{\frac{1}{2}\log\left(1+\frac{g^{2}P}{\sigma^{2}}\right),{\left[\frac{1}{2}\log\left(1+\frac{g^{2}P}{\sigma^{2}}\right)-\frac{1}{2}\log\left(1+\frac{g_{e}^{2}P}{\sigma_{e}^{2}}\right)\right]}^{+}+\frac{1}{2}\log\left(2\pi e\sigma^{2}\right)\right\}. \end{array}$$

Second, in [17], it has been shown that for the discrete memoryless non-degraded wiretap feedback channel, a lower bound $R_{s-f}^{***}$ on the secrecy capacity, which is constructed by the improved secret key-based feedback scheme, is given by:

(20) $$\begin{array}{cc} & R_{s-f}^{***}=\max_{P(x)}\min\left\{{\left[I\left(X;V,Y\right)-I\left(X;Z\right)\right]}^{+}+H\left(Y|X,Z\right),I\left(X;Y\right)\right\}, \end{array}$$

where the joint distribution is denoted by:

(21) $$\begin{array}{cc} & P(v,x,y,z)=P(v|x,y)P(y|x)P(z|x)P(x). \end{array}$$

Then, substituting X∼N(0,P), V=X+Y, and (1) into (20), a lower bound $R_{s-f}^{non**}$ on the secrecy capacity $C_{s-f}$ is obtained, and it is given by the following Corollary 3.

**Corollary** **3.** *A lower bound $R_{s-f}^{non**}$ on the secrecy capacity $C_{s-f}$ of the non-degraded Gaussian wiretap feedback channel is given by:*

(22) $$\begin{array}{cc} & R_{s-f}^{non**}=\frac{1}{2}\log\left(1+\frac{g^{2}P}{\sigma^{2}}\right). \end{array}$$

**Proof.**  First, substituting X∼N(0,P), V=X+Y, and (1) into (20), we have:

(23) $$\begin{array}{cc} & R_{s-f}^{non**}=\min\left\{\frac{1}{2}\log\left(1+\frac{g^{2}P}{\sigma^{2}}\right),{\left[\frac{1}{2}\log\left(2\pi eP\right)-h\left(X|X,Y\right)-\frac{1}{2}\log\left(1+\frac{g_{e}^{2}P}{\sigma_{e}^{2}}\right)\right]}^{+}+\frac{1}{2}\log\left(2\pi e\sigma^{2}\right)\right\}. \end{array}$$

Next, note that the conditional differential entropy term h(X|X,Y) in (23) equals −∞. Now, substituting h(X|X,Y)=−∞ into (23), we can conclude that:

(24) $$\begin{array}{cc} & {\left[\frac{1}{2}\log\left(2\pi eP\right)-h\left(X|X,Y\right)-\frac{1}{2}\log\left(1+\frac{g_{e}^{2}P}{\sigma_{e}^{2}}\right)\right]}^{+}+\frac{1}{2}\log\left(2\pi e\sigma^{2}\right)=\infty , \end{array}$$

and this leads to the fact that $R_{s-f}^{non**}=\frac{1}{2}\log\left(1+\frac{g^{2}P}{\sigma^{2}}\right)$. The proof is complete. □

From Corollary 3, we see that $R_{s-f}^{non**}$ is exactly the same as the secrecy capacity given in Theorem 1, which indicates that for the non-degraded Gaussian wiretap feedback channel, the improved secret key-based feedback scheme performs as well as the SK feedback scheme, and both of them achieve the secrecy capacity of this non-degraded model.

**Figure 4:**
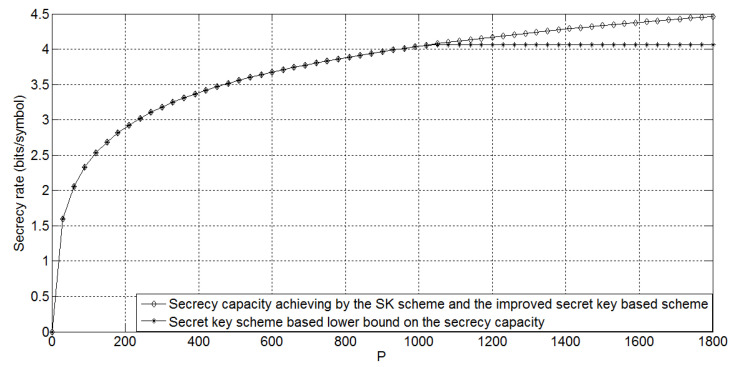
The capacity results on the non-degraded Gaussian wiretap feedback channel for g=0.9, $g_{e}=0.7$, $\sigma^{2}=3$, $\sigma_{e}^{2}=10$, and *P* taking values in [0,1800].

The following Figure 4 shows the comparison of the SK scheme, secret key-based scheme, and the improved secret key-based scheme for g=0.9, $g_{e}=0.7$, $\sigma^{2}=3$, $\sigma_{e}^{2}=10$, and *P* taking values in [0,1800]. It is easy to see that the performance gap between the secret key-based scheme and other two schemes is increasing while the transmitting power *P* is increasing.

**Figure 5:**
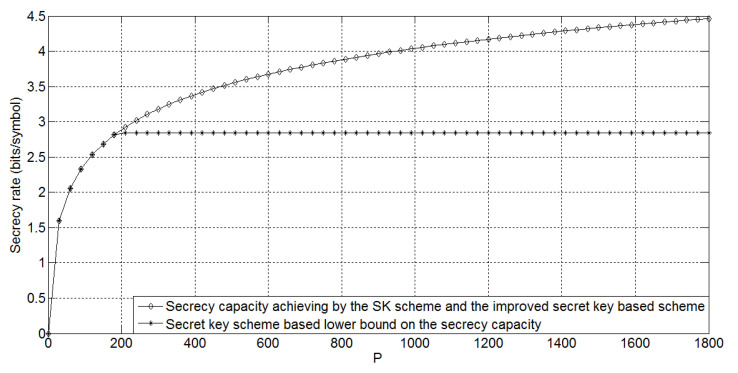
The capacity results on the non-degraded Gaussian wiretap feedback channel for g=0.9, $g_{e}=0.7$, $\sigma^{2}=3$, $\sigma_{e}^{2}=0.1$, and *P* taking values in [0,1800].

In addition, the following Figure 5 shows the comparison of the SK scheme, the secret key-based scheme, and the improved secret key-based scheme for g=0.9, $g_{e}=0.7$, $\sigma^{2}=3$, $\sigma_{e}^{2}=0.1$, and *P* taking values in [0,1800]. Comparing Figure 5 with Figure 4, we can conclude that when the eavesdropper’s channel noise variance is decreasing and the transmitting power *P* is increasing, the performance gap between the secret key-based scheme and other two schemes is increasing.

## 4. Conclusions

In this paper, we determined the secrecy capacity of the non-degraded Gaussian wiretap feedback channel and showed that it equals the channel capacity of the same model without the secrecy constraint. Moreover, we compared the performances of the SK scheme, the secret key-based scheme, and the improved secret key-based scheme in the Gaussian wiretap feedback channel and showed that for the non-degraded case, the improved secret key-based scheme performs as well as the SK scheme, and both of them are better than the secret key-based scheme. Numerical results indicated that the performance gap between the secret key-based scheme and other two schemes was increasing while the eavesdropper’s channel noise variance was decreasing and the transmitting power *P* was increasing.
